# Novel insights into courtship and mating behavior of *Anastatus japonicus* enhance pest control and mass-rearing efficiency

**DOI:** 10.1038/s41598-025-13574-3

**Published:** 2025-10-09

**Authors:** Muhammad Yasir Ali, Donato Romano, Zijian Song, Ana. L. Favoreto, Khalid Ali Khan, Jin-Ping Zhang, Feng Zhang

**Affiliations:** 1 MARA-CABI Joint Laboratory for Bio-safety, Institute of Plant Protection, Chinese Academy of Agricultural Sciences, Beijing, 100193 China; 2 CABI, Beijing, 100081 China; 3 The BioRobotics Institute and Department of Excellence in Robotics and AI, Sant’Anna School of Advanced Studies, 56127 Pisa, Italy; 4 Key Laboratory of Soybean Disease and Pest Control (Ministry of Agriculture and Rural Affairs), College of Plant Protection, Jilin Agricultural University, Changchun, 130118 China; 5 Departamento de Proteção Vegetal, Faculdade de Ciências Agronômicas, Universidade Estadual Paulista (UNESP), Botucatu, SP 18610-034 Brazil; 6 Departamento de Entomologia/BIOAGRO, Universidade Federal de Viçosa, Viçosa, MG 36570-90 Brazil; 7 Center of Bee Research and its Products (CBRP) and Research Center for Advanced Materials Science (RCAMS), King Khalid University, P. O. Box 9004, 61413 Abha, Saudi Arabia; 8 Applied College, King Khalid University, P. O. Box 9004, 61413 Abha, Saudi Arabia

**Keywords:** Biological control, Parasitoid wasp, Mating behavior, Monandry, Fecundity, Chemical biology, Ecology, Physiology, Zoology

## Abstract

The solitary egg endoparasitoid, *Anastatus japonicus* (Hymenoptera: Eupelmidae), holds substantial potential for effectively controlling hemipterous and lepidopterous pests. The present study endeavors to elucidate the courtship and mating behavior of this parasitoid, as a comprehensive understanding of female and male mating status and its implications on offspring production remains inadequately documented. Courtship and mating behavior process impacted by food, age, host, body size, virgin and mated both sexes were monitored by direct observation, while fecundity and female proportion of virgin and mated females were tested in Petri dishes. During courtship, only males make physical contact with the antennae and thorax-abdomen regions of females. Mating success was quicker at younger age of both sexes (i.e. < 24 h old), and higher when *A. japonicus* males approached the female from the left side (right biased) and preferential turning on the right (left biased) to attempt copula resulting in monandry and polygyny behavior in female and male, respectively. Females prefer to mate with virgin males over mated, and honey fed males were preferred over starved ones. *Anastatus japonicus* unmated females are haploid and produced only males, however mated females are diploid and produced both progenies. Furthermore, females showed synovigenic strategy and produce more offspring numbers (11–14) and females’ proportion (83–92%) at older age (10–30 d old) rather than younger aged (0–5 d, offspring number: 5–10; female proportion: 62–72%). Female wasps that mated with already mated males produce a smaller proportion of females (with virgin male: 61.88%, mated male: 37.17%), exhibiting possible sperm depletion effect. It is highly expected that a tailor-made large-scale rearing system of *A. japonicus* will be developed to optimize mating success and female-biased progeny production to fully utilize its reproduction potential and to ultimately improve mass-rearing efficiency.

## Introduction

The utilization of natural enemies for insect biological control has become an integral component of various pest management programs. Enhancing the effectiveness and population sizes of these natural enemies, particularly through mass rearing techniques, has been suggested as a key strategy^1^. Reproductive behavior and fecundity are thus essential to help develop effective mass rearing system. Parasitoid courtship typically encompasses a series of behaviors, including antennal tapping, antennal grooming, trail-following, body or abdomen vibration, wing fanning, and mounting^2^. Understanding courtship and mating behaviors in parasitoids is of paramount importance, as the success of parasitoids in biological control programs hinges on their ability to locate mates and elevate the female sex ratio, thus ensuring both insect reproduction and population establishment^3^. Given the critical role of courtship behavior in parasitoid reproduction, another factor that may influence mating efficiency is lateralization—the asymmetric processing of sensory and motor signals^4^.

Lateralization (i.e., right-left imbalance of brain and behavior) is an intriguing concept associated with brain function^5^. It may assist in improving brain efficiency in cognitive tasks that involve both hemispheres, such as processing multiple types of information at the same time^6^. The majority of study focuses on lateralized attributes in vertebrate animals^7^. However, several studies also pay attention on insect species’ asymmetries in the brain at individual and population level^8,9^. The lateralization of courtship and mating behavior has been studied in several insect species, including a calliphorid fly^10^, hemipteran bugs^5,11^, mosquitoes^12^, stored product beetles^8,13^ and tephritid fly^14^. However, currently negligible amount of information is known about the lateralization of courtship and mating behavior in insect species of the order hymenoptera, particularly no information on egg parasitoids.

Hymenopteran parasitoids with a haplo-diploid sex determination system can reproduce asexually, albeit yielding only male progeny^15^. Consequently, newly emerged female parasitoid wasps face a crucial decision: to either promptly seek out hosts, resulting in exclusively male offspring, or to invest time and energy in mating before ovipositing, thus enabling the production of both males and females^16^. Generally, the latter option is favored for population expansion^17,18^. Additionally, a preferred trait in potential mates for both males and females is virginity, alongside considerations of body size^19^. An individual’s own mating status or self-perceived mate value can also influence its sexual behavior^20^.

*Anastatus japonicus* Ashmead (Hymenoptera: Eupelmidae) belongs to one of the most widely distributed genera of Eupelmidae worldwide^21,22^. It is a known egg parasitoid of lepidopterans like *Lymantria dispar* (L.)^23,24^, *Actias selene ningpoana* (Felder), *Antheraea pernyi* (Guerin-Meneville), *Caligula japonica* (Moore), *Dendrolimus punctatus* (Walker), *Malacosoma neustria testacea* (Motschulsky), and *Odonestis pruni* (Linnaeus)^1,25^, and has been reported in various studies as parasitizing several other significant pests, including hemipterans such as *Tessaratoma papillosa* Drury (Hemiptera: Pentatomidae)^26^, *Halyomorpha halys* (Stål) (Hemiptera: Pentatomidae)^27^. Importantly, *A. japonicus* was introduced to North America in the early 1900s to serve as a biological control agent against *L. dispar*^1,24^, demonstrating its effectiveness in controlling the target species*.* This parasitoid, initially misidentified as *A. bifasciatus*, also inhabits Canada and the United States (Nearctic region)^22^. The efficient utilization of *A. japonicus* for managing hemipteran and lepidopteran pests necessitates an in-depth understanding of its reproduction, including courtship and mating behaviors, which are pivotal aspects of its biology^28^. Nevertheless, studies on the reproductive biology of this species have been relatively limited, with variables such as age, body size and nutrition known to influence mating and progeny production success^19,29^.

The maternal age of *A. japonicus* leads to male-biased offspring production, a limitation to fully utilize its potential in the biological control of insect pests^22^. The primary objective of this study is to assess fecundity behavior of female at different critical ages where fluctuations in sex ratio occurs leading to male biased offspring. We hypothesize that *A. japonicus* females exhibit synovigenic behavior—continuously maturing new eggs during adulthood—and thus require time for ovarian development to maximize offspring production, particularly female progeny. Additionally, sperm limitation in female spermatheca is a factor leading to increased male production. Thus, we hypothesize that mated females may mate again when their spermatheca reaches a state of diminished sperm. Furthermore, the present study investigates courtship and mating behavior to understand how males and females adjust their mating behavior, in particular, whether females exhibit a preference for mating with virgin males over mated ones. These insights are critical for optimizing rearing and field-release protocols and for comprehending the underlying mechanisms governing mating dynamics and population size.

## Results

### Observation of courtship and mating behavior of *A. japonicus*

Male *A. japonicus* were active during courtship, while females stayed passive with low response movements. The released male parasitoid wasps ran around the arena in the Petri dish to identify the area where they might begin courtship as soon as they found the female, but females kept stationary or, on occasion, surveyed the arena.

The time of male (♂) to find the female (♀) depends upon its own and ♀ age (F_203, 6_ = 975.147, *P* > 0.05). Newly emerged ♂ and ♀ take significantly shorter time (27.9 s) as compared with 24 h (29.53 s), 48 h (32.42 s) and 5 d old ones (40.99 s) (F_116, 3_ = 254.143, *P* < 0.05) (Table 1; first row of small alphabets). On the other side, the ♂ takes longer time to sense aged ♀ (Table 1; second row of capital alphabets), however the aged ♂ did not take longer time to sense ♀ (F_116, 3_ = 1539.74, *P* < 0.05) (Table 1; third row of lower alphabets).

**Table 1:** Time (mean ± SE) required for *A. japonicus* (Hymenoptera: Eupelmidae) males to locate females.

Age of male status	Age of female status	Time (seconds) *
Newly emerged	Newly emerged	27.90 f D w
Newly emerged	24-h-old	31.05 de C
24-h-old	Newly emerged	29.53 e x
Newly emerged	48-h-old	36.31 c y
48-h-old	Newly emerged	32.42 d B
Newly emerged	5-d-old	61.47 a z
5-d-old	Newly emerged	40.99 b A

*Time calculated in seconds. Different alphabets denote significance difference; first column in small letters: overall significance among all treatments while capital and lower alphabets in second and third column represents among different aged male and female, respectively (ANOVA; *p* < 0.05).

The ♂ approached ♀, moved from his position to clock or anticlock wise towards ♀ antennae significantly differ between four different ♂ and ♀ treatments (K-W; H = 98.641, df = 3, *P* < 0.05), ♂ touched ♀ with its antennae (K-W; H = 111.22, df = 3, *P* < 0.05), intertwined its antennae with that of the ♀ with a slight back and forth movement. Afterward, the ♂ moved again in a clock or anticlockwise position, followed by chasing as a mating attempt, which did not differ between mated or unmated ♂ with mated ♀ (*P* > 0.05) but significantly differ (*P* < 0.05) between virgin ♀ with virgin and mated ♂ (K-W; H = 52.35).

**Fig. 1:**
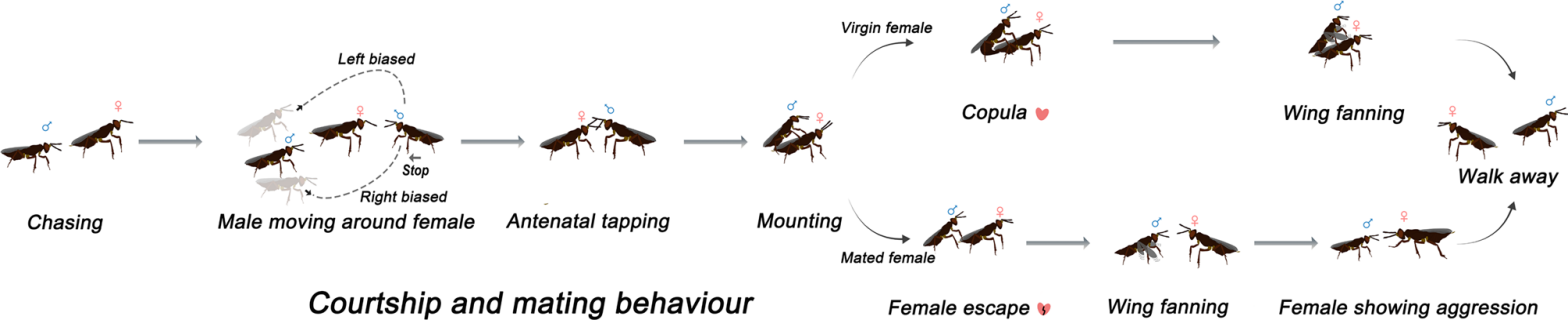
Ethogram depicting the courtship and mating sequence of the egg parasitoid wasp *A. japonicus*. (n = 15 replications for virgin and 15 for mated female).

In the case of nonreceptive/already mated ♀, the duration of ♂ moving towards ♀, antennae–antennae touching, mounting, and number of bouts significantly increased which seems aggressive response by ♀ and does not let ♂ to go ahead for copulation. Furthermore, in case the ♂ succeeded in climbing at ♀ then ♀ immediately escaped away. In case of successful mating, the ♂ right after mating started vibrating while standing on the thorax of ♀, and hereafter ♂ and ♀ walked off. However, in already mated ♀ cases, the ♂ started moving around ♀ even after unsuccessful bouts. Lastly, the copulation duration had no significant difference (M-W; U = 331, df = 1, *P* = 0.318), however, increased in mated ♂ as compared with virgin ones (1.24 s vs. 1.18 s) (Table 2; Fig. 1).

**Table 2:** Mating behavior, frequency and duration of *Anastatus japonicus* males.

Category	Behavior	Time (s)			
		Virgin male × Virgin female	Virgin male × Mated female	Mated male × Virgin female	Mated male × Mated female
Courtship	♂ Moving around ♀	1.28 ± 0.03 d	3.18 ± 0.09 b	1.73 ± 0.09 c	4.00 ± 0.08 a
	Antennae–antennae	2.02 ± 0.03 d	3.51 ± 0.07 b	2.57 ± 0.04 c	5.40 ± 0.09 a
Precopulatory	Mounting (seconds)	1.21 ± 0.02 c	0.94 ± 0.05 b	1.40 ± 0.05a	0.98 ± 0.04 b
	Number of bouts	1	3–5	1	3–4
	Aggression by ♀ (behavioral act)	Female receptive. In few cases show aggression when male touch his aedeagus at wrong position	Female nonreceptive. Reject-partner by showing extreme aggression	Female receptive. In few cases show aggression when male touch his aedeagus at wrong position	Female nonreceptive. Reject-partner by showing extreme aggression
	Aggression by ♂	None	Male shows wing fanning as female reject mating	None	Male shows wing fanning as female reject mating
Copulation	Copulation (seconds)	1.18 ± 0.02 a	None	1.24 ± 0.03 a	None
	Mating	Yes	None	Yes	None
Postcopulatory	Wing fanning	Male starts wing fanning right after copulation while standing at the thorax of female	No	Male starts wing fanning right after copulation while standing at the thorax of female	No
	Walk away	After successful mating male and female walk away	Keep moving around until female shows extreme aggressiveness	After successful mating male and female walk away	Keep moving around until female shows extreme aggressiveness

Different alphabetics represent significant difference among treatments.

**Fig. 2:**
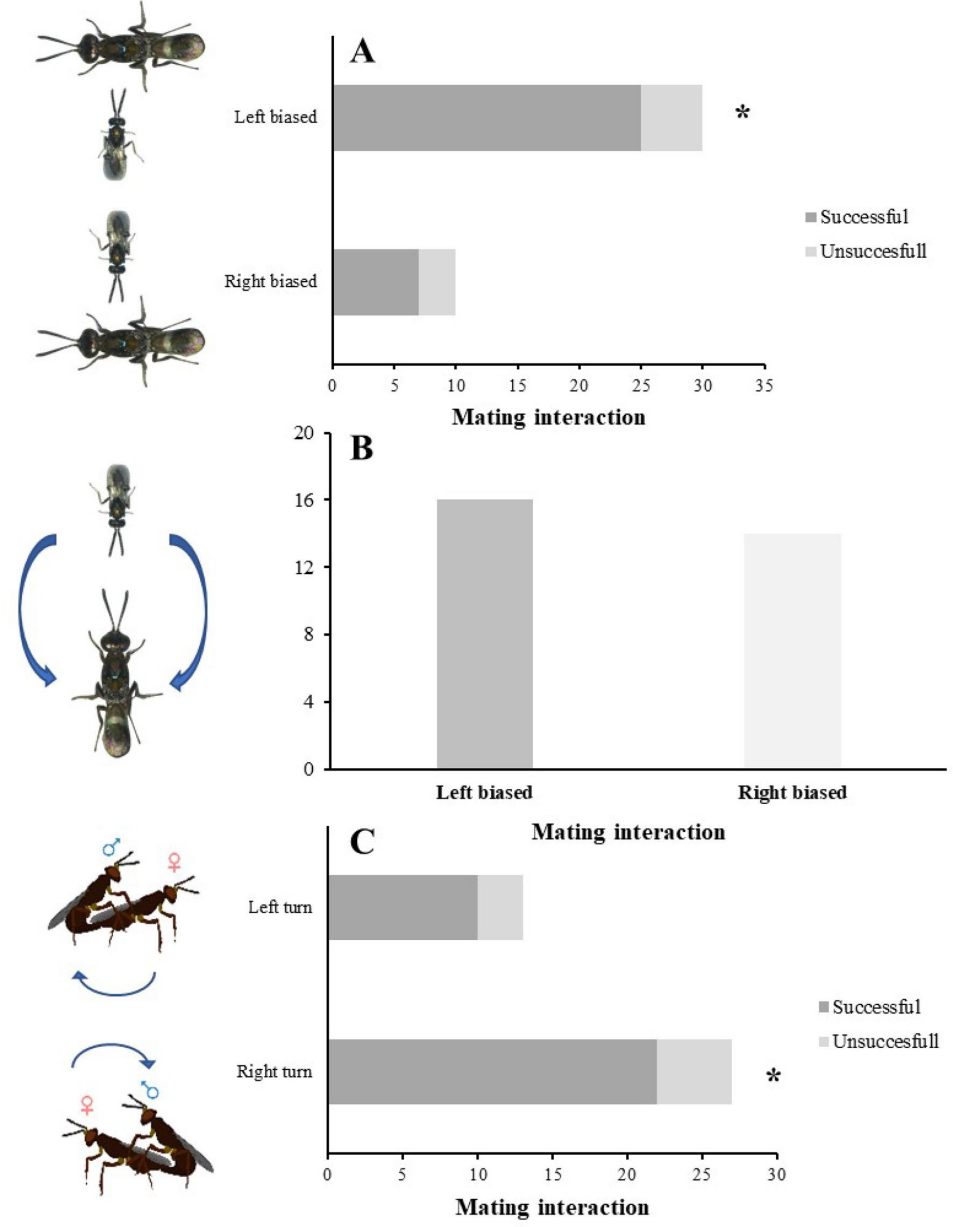
Mating success of *A. japonicus* males showing (**a**) left or right-biased approaches to the female, and (**b**) face to face interaction display (**c**) left or right-biased turning displays; asterisks (χ^2^ test with Yates’ correction) indicate a significant difference left and right-biased acts.

In addition, mating success was higher when *A. japonicus* males approached the female from the left side during sexual interactions (M-W; U = 350.09, df = 1, *p* < 0.05), while approaches from the right side did not affect significantly mating success (M-W; U = 269.32, df = 1, *p* > 0.05) (Fig. 2A); however, no significance when comes face to face in terms of right or left biased (M-W; U = 165.45, df = 1, *p* > 0.05) (Fig. 2B). Eventually the preferential turning on the right to attempt copula led to higher number successful males in mating (M-W; U = 270.42, df = 1, *p* < 0.05), compared to left-biased turning males (M-W; U = 190.29, df = 1, *p* > 0.05) (Fig. 2C). Moreover, other influence in mating were the body sizes, larger (0.063 ± 0.01 mm) and smaller males (0.044 ± 0.01 mm) were calculated in terms of hind tibia lengths. When the relative difference between the larger and smaller males for mating was considered, larger males had a considerably higher chance of winning competitions (Chi square χ^2^ = 19.200, df = 1, *P* ≤ 0.0001) (90%).

### Mating frequency and process sustain time of *A. japonicus* at different age

**Fig. 3:**
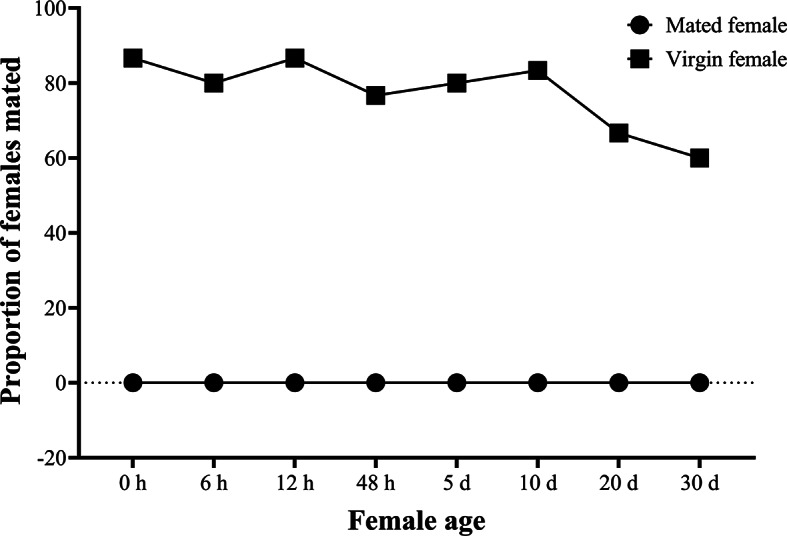
Mating proportion of virgin and mated females with virgin males at different time intervals completed during 30 min.

**Fig. 4:**
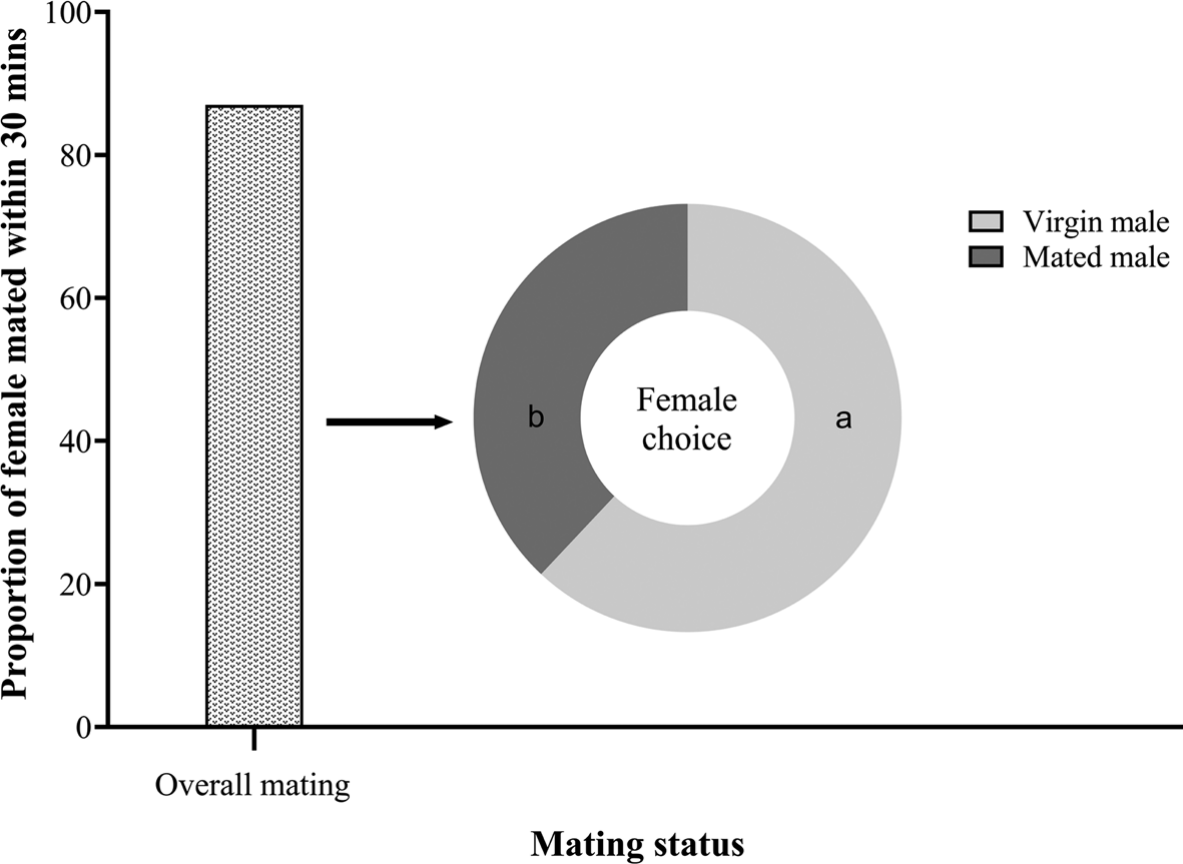
The bar graph represents the proportion of overall mating, while pie graph further represents choice of mating success within thirty minutes of duration by virgin female *A. japonicus*, respectively. Different letters denote significant differences.

*Anastatus japonicus* females- mate only once hence exhibiting monandry mating behavior (Fig. 3). In total thirty pairs were observed with no successful copulations in mated female, although most males tried to mount the mated female and then repeated the cycle after rejection by the female. Our observations showed that the majority of males, regardless of size or age, were attracted to virgin and mated females, however, only virgin females allowed males to mount. The successful rate of mating for virgin female was 87% within thirty minutes (Chi square χ^2^ = 16.13, df = 1, *P* < 0.05). Females showed preference for virgin males over mated males (Chi square χ^2^ = 1.385, df = 1, *P* < 0.05) (Fig. 4). The first mount occurred after 38.01 ± 10.28 s when the female was exposed to the virgin male, and after 41.73 ± 12.01 s when it was exposed to the mated male, and there was no statistically significant difference (M-W; U = 146, df = 1, *P* > 0.05). The probability of successful mating was unaffected by the color of markings applied to males.

**Fig. 5:**
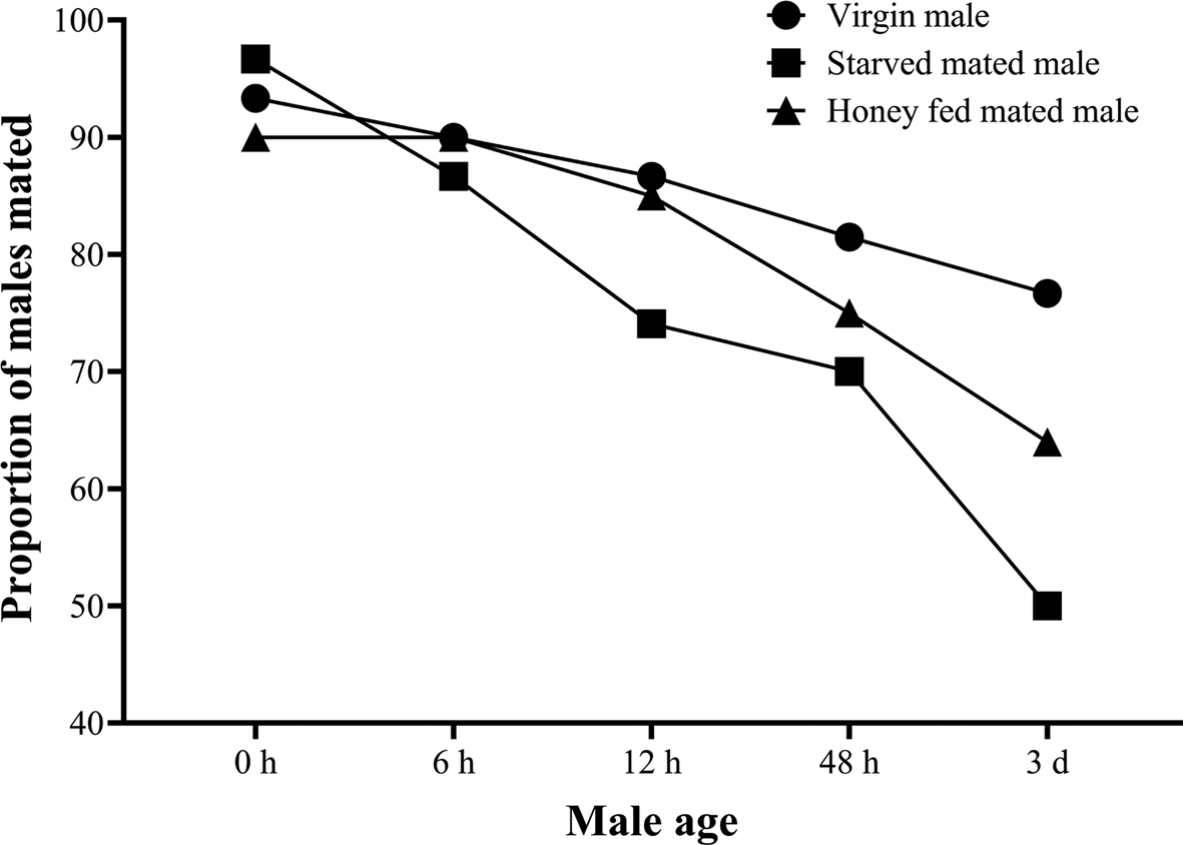
Mating proportion of virgin, mated and honey fed mated males with virgin females at different time intervals completed during 30 min.

*Anastatus japonicus* males can mate multiple times throughout their life span exhibiting polygyny mating behavior (Fig. 5). Moreover, the honey fed mated male showed higher mating proportion as compared with starved mated one (M-W; U = 121.440, df = 1, *P* < 0.05) however overall virgin males showed highest mating proportion among them (Wald; χ^2^ = 1.993, df = 3, *P* > 0.05) (Fig. 5). The mating proportion was higher in younger and mid-age wasps (from 0 h to 10 d in female; from 0 h to 48 h in males) than older age ones (11–30 d old females; 72 h old males) (female: Wald; χ^2^ = 150.32, df = 7, *P* < 0.05) (male: Wald; χ^2^ = 212.42, df = 4, *P* < 0.05) (Figs. 3, 5).

### Influence of age on progeny production within 24 h after mating

**Fig. 6:**
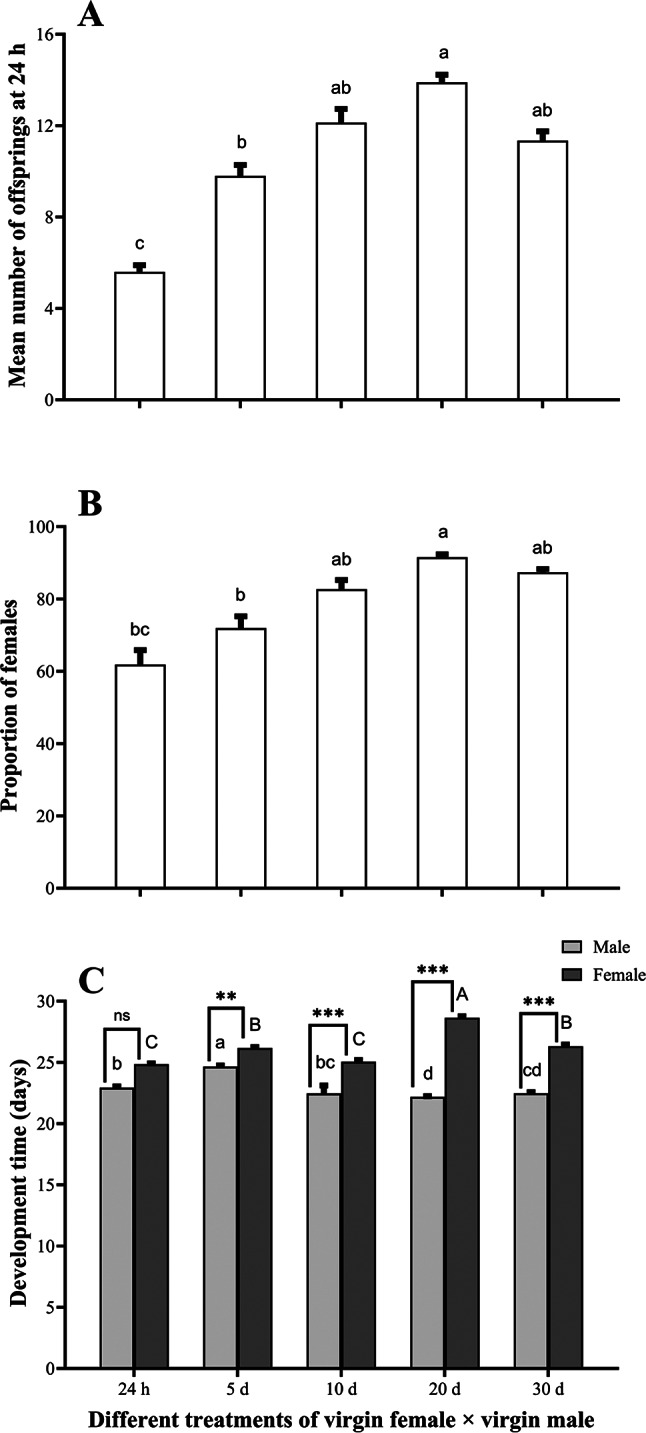
Mean (± SE) (**A**) total number of progenies at 24 h exposure, (**B**) proportion of females (**C**) male and female development time of *A. japonicus* on five different female age treatment setups. Small and capital letters denote significant differences in male and female development time at different treatments, respectively (*P* < 0.05). N.S denotes no significant difference while ** and *** denote *p* < 0.001, *p* < 0.0001, respectively by independent sample *t* test.

*Anastatus japonicus* unmated females produced only males, however mated females produced both progenies. Twenty-four hours old females produced significantly smaller number of offspring (K-W; H = 86.162, df = 4, *P* < 0.05; Fig. 6A) and female proportion than on subsequent days (Wald; χ^2^ = 125.440, df = 4, *P* < 0.05; Fig. 6B). Mated females’ fecundity upon reaching maturity of ovary i.e. at fifth, tenth, twentieth and thirtieth day showed slight difference (K-W; H = 168.440, df = 4, *P* < 0.05; Fig. 6A). Similarly, the proportion of sex ratio in all five treatments did not differ statistically (Wald; χ^2^ = 95.440, df = 4, *P* > 0.05; Fig. 6B). However, no significant difference was observed in the development time of males (K-W; H = 121.440; df = 4, *P* > 0.05) and female (K-W; H = 480.190; df = 4, *P* > 0.05) at different time intervals but male emerged ~ 1.5–2 d earlier than female at each treatment (Fig. 6C). Significant difference was observed among male and female’s development time via independent sample *t* test except 24 h treatment (*t* = − 9.81, *P* = 0.5). Highest significant difference was noted among male and female at 10th (*t* = − 0.83, *P* < 0.0001) 20th (*t* = − 18.59, *P* < 0.0001) and 30th (*t* = − 11.91, *P* < 0.0001) day treatment however slightly lower significance was found at 5th day treatment development time (*t* = − 10.29, *P* = 0.0001).

**Fig. 7:**
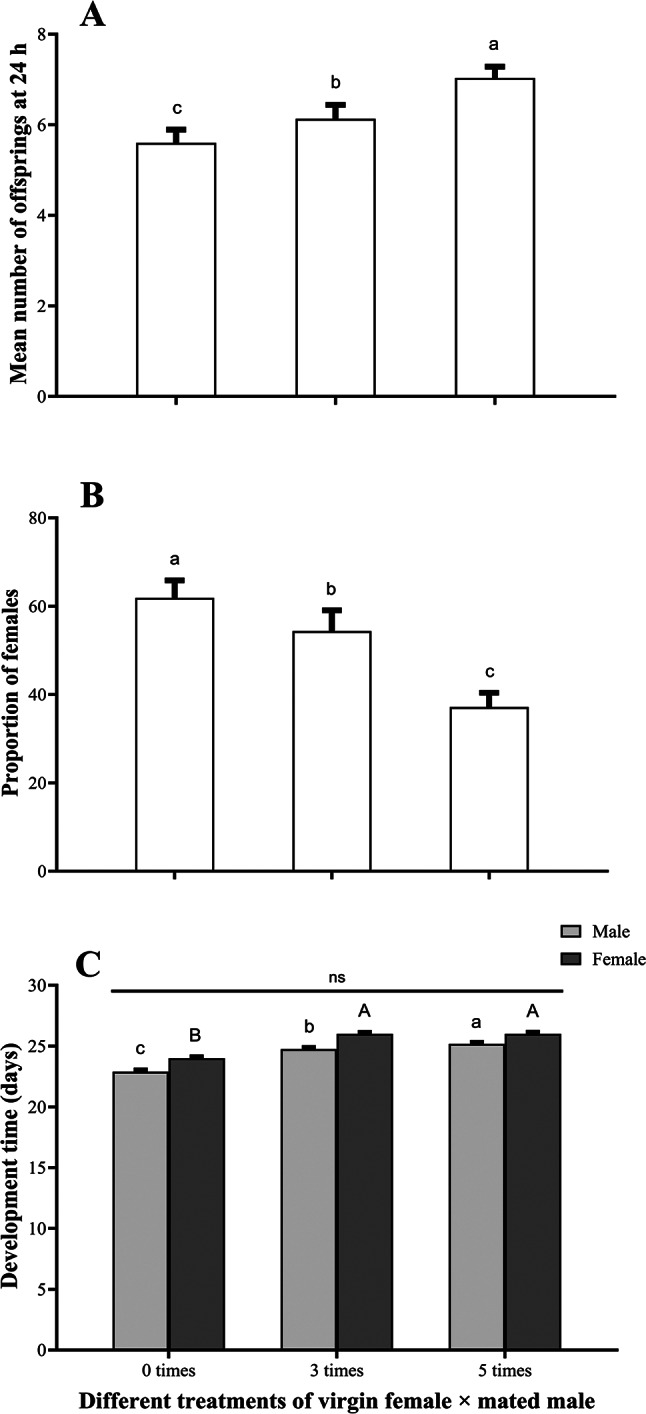
Mean (± SE) (**A**) total number of progenies at 24 h exposure, (**B**) proportion of females (**C**) male and female development times of *A. japonicus* on three different male mating treatment setups. Small and capital letters denote significant differences in male and female development time at different treatments, respectively (*P* < 0.05). N.S denotes no significant difference among male and female development time—independent sample *t* test.

Females produced the highest daily fecundity when mated with five times mated male (ANOVA; F _2, 87_ = 6.517, *P* < 0.05; Fig. 7A) while maximum female proportions were produced by females mated with virgin males (Wald; χ^2^ = 89.78, df = 2, *P* < 0.05; Fig. 7B), suggesting possible sperm depletion for already mated males. There was no significant difference between male (K-W; H = 78.911, df = 2, *P* > 0.05) and female (K-W; H = 94.049, df = 2, *P* > 0.05) development times at different time intervals, however, male emerged ~ 1–1.5 d earlier than female at each treatment (Fig. 7C). In addition, independent sample *t* test (*P* > 0.05) exhibits that there is no significant difference among the development time of male and female.

## Discussion

Male *A. japonicus* initiated courtship by antennal tapping on the female’s antennae and/or thorax-abdomen, a recognition behavior common to many parasitoid wasps—including *Leptomastidea abnormis*, *Anagyrus pseudococci* and *Psyllaephagus bliteus*—that facilitates mate identification and subsequent mating sequences^30^. Courtship behavior can stimulate female receptivity, as suggested in *Ibalia japonica* Matsumura (Hymenoptera: Ibaliidae) and *Sphex ingens* Smith (Hymenoptera: Sphecidae), where it reduces courtship duration and increasing reproductive success^31,32^. For instance, *P. bliteus* females aged 48 h older become receptive to male wing fanning and abdomen movements, a preparatory behavior also seen in the parasitoids *L. abnormis* and *A. pseudococci*^33^. These females respond by dorsally bending their abdomen and spreading their wings, a pre-copulatory posture similarly exhibited by *A. japonicus*^34,35^. Additionally, *A. japonicus* prefer to mate shortly after emergence, mirroring patterns in *Cleruchoides noackae* Lin & Huber (Hymenoptera: Mymaridae), where female mate immediately post-emergence^36^. However, female rejection of mating attempts is a common behavior and may occur for various reasons (i.e. mate choice, timing and reproductive state), wherein they walk or fly away from the male^30^, such as that observed for *P. bliteus* and *A. japonicus*. However, *S. ingens* females are more aggressive, partially opening their mandibles and tilting the body vertically^37^.

The results of this study further demonstrated that the age of the male and female plays a crucial role in the duration of locating a mate. Our results are consistent with *Diatraea considerata* and *Copitarsia decolora* findings that the younger males and females took significantly shorter time to find mate^38,39^. Our investigations revealed that virgin males are quicker to perform whole courtship, however in other studies mated males are observed to be quicker towards dead females^40^. On the other hand, mated males are perhaps less active than virgins due to several reasons, i.e. fatigue^41^.

The lateralized courtship in *A. japonicus* demonstrates conserved neurological specialization seen across insects^42^, yet reveals a distinctive left-side approach that contrasts markedly with the right-biased behaviors documented in *Trichogramma* spp.^4^. This previously unreported directional asymmetry in Eupelmidae may represent a family-specific adaptation, highlighting the need for comparative studies across related taxa. Lateralized results have critical implications for mass rearing, as artificial environments may disrupt natural lateralized behaviors—a phenomenon shown to reduce mating success by 15–25% in comparable systems^43^. We propose that incorporating three-dimensional structures in rearing containers could better accommodate these turning behaviors, potentially improving production efficiency while maintaining natural mating dynamics. Future studies should examine whether preserving these lateralized patterns enhances field performance^5^.

The effect of female status was that virgin and mated females were contacted and mounted about equally by males. However, after being mounted, virgin and mated females were usually receptive and non-receptive to copulation, respectively. *Anastatus japonicus* female exhibits a body size preference, is one of the most apparent life-history traits of organisms^44^ and is usually positively correlated with many fitness components in parasitoids^45,46^. As mating is directly related to male fitness, access to female mates is important to males^47^. Body size has independent and striking effects on the probability of winning mating. It is likely that in environments with few mating opportunities, the potential benefits of winning can exceed the costs of fatal combat^48^.

The female *A. japonicus* is monandrous (mate only once) and male shows polygynous (mate several times) behavior. The majority of all rematings in other insects occurred when the female failed to produce female offspring from her first mating. But this was not observed in our case as all mated females were able to produce both sex offspring during their oviposition periods. Female *A. japonicus* produced less number of offspring and female numbers at the age of 24 h as compared with day 5, 10, 20, and 30, showing that the wasps emerged with less/no mature eggs in their ovaries, and the number of mature eggs increased rapidly thereafter throughout her life cycle, a process termed synovigeny^49,50^. During the synovigenic reproductive process, male insects may transfer specialized ejaculate components—such as seminal fluid proteins (SFPs) in *Aedes aegypti*—that stimulate oviposition by modulating female post-mating physiology^51^. However, maternal age has no effect on progeny production after the ovary maturation.

Female wasps that mated with non-virgin males decreased the production of daughters (fertilized eggs), suggesting that sperm-limited males provide an insufficient amount of sperm to the females^52^. Studies on other parasitoid wasps have reported that females under sperm limitation indiscriminately re-mate and recover the number of stored sperm^18,53^. However, female *A. japonicus* typically did not engage in re-mating. Moreover, a species that has been selected to strongly favor monandry may still re-mate under certain unusual conditions, such as extreme temperatures^54^. At these extremes, sperm viability within the female’s spermatheca decreases, making it immotile. Consequently, females are unable to fertilize their eggs, resulting in the production of only male offspring. This aspect warrants further exploration in *A. japonicus*. In the case of *A. japonicus*, mounted virgin-females were more likely to become receptive to copulation by a virgin male than by a mated male.

There are several possible explanations for why both virgin and mated males actively pursue, mount, and court already mated females, even though these females do not engage in multiple mating^55^. Males may be not constrained by limitations in time or energy, making failed attempts inconsequential. Alternatively, male encounters with mated females may be rare, for instance, if females typically disperse after mating^40^. There may be a rewarding aspect to mating which mated males learn to associate with female pheromones^56^ which has been studied in the confamilial *N. vitripennis*^57^.

In this study, females exhibited a mating preference for virgin males. Previous research suggests that virgin males may have higher levels of seminal fluid proteins, which could influence female choice^58^. In addition, a female mated to already mated male produces a substantially lower proportion of daughters because mated males have a lower amount of sperm so she avoids mating with mated males^59^. Furthermore, across insects, when mating status affects sexual interactions, there tends to be a preference by males and females for virgins^60,61^ and greater sexual responsiveness by virgins, even in polyandrous and polygynous species^62^. Furthermore, another evidence of female choosiness for virgin males in parasitoids was documented in *N. vitripennis*, in which males release lower amounts of a sex pheromone as their sperm stores decrease and females are more attracted to males that release higher amounts of the pheromone^63^.

*Anastatus japonicus* males and females are solitary parasitoids, i.e. males and females do not emerge simultaneously. Mating is a social behavior and male of this natural enemy copulates with various partners however females mate only once. Moreover, males and females prefer to mate right after emergence but could delay up to 48 h. Females of this parasitoid emerge with immature eggs and present parasitism with lower female proportion however female proportion increases as the insect goes old, mainly, after they are 5 d old. Weekly 2–3 times honey feeding is necessary as the honey fed males are the priority for female to mate and their healthy sperm would lead to produce more female numbers. Unmated females produce only males so individuals of both sexes should remain together in laboratory rearing for at least 48 h to ensure that they mate and ultimately females are able to produce both offsprings. Males emerge before the females and wait for her to come out by standing close to the eggs for copulation exhibiting the involvement of some unique sex pheromone which needs to be explored in upcoming studies. While *A. japonicus* oviposition is triggered by artificial host (*A. pernyi*) chemicals (unpublished-data), whether natural host (*H. halys*) derived semiochemicals elicit stronger responses is unknown—a critical gap for improving biocontrol efficiency.

## Material and Methods

### Insect culture

*Anastatus japonicus* were originally obtained from parasitized *Halyomorpha halys* eggs collected in a peach orchard in Beijing, China (N 40° 020 0600, E 116° 120 4100). The parasitoid colony was maintained in transparent acrylic rearing cages (25 cm × 25 cm × 25 cm) since 2015. Parasitoids were fed honey twice a week and held under laboratory condition of 25 ± 3 °C, 60 ± 5% relative humidity (RH), and 16 h:8 h light/dark photoperiod. To maintain laboratory colony of *A. japonicus*, frozen eggs of *A. pernyi* (Fucai *Trichogramma* Production Professional Cooperative, Xifeng County, Liaoning Province, China) were provided to the parasitoid for continuous rearing. Different aged adult parasitoids were collected according to the need of the experiments. All bioassays were done in an incubator (BluePard Series, Yiheng Technology Company, Shanghai, China) at 25 ± 1 °C, 70 ± 5% RH, and 16 h:8 h light/dark photoperiod. Parasitoids identification was already done in earlier study through morphological and CO1 techniques^21^. All of the experiments performed in this study were replicated 30 times.

### Observation of courtship and mating behavior of *A. japonicus*

*Anastatus japonicus* courtship and mating behavior were analyzed through direct observation under a stereoscopic microscope ZEISS, Leica Application Suite (version 2.1.0). A newly emerged virgin male and female (< 24 h old) were paired and introduced to a Petri dish (diam. = 6 cm) alongside 20% honey solution cotton wick as a food source. This procedure allowed the insects to approach each other and, if accepted by the female or male, to mate. Each pair of wasps was observed for a maximum of 30 min or until the successful courtship ended with the male and female physically separating after copulation. Successful rate of mating in the observed 30 min, latency to courtship, courtship duration, precopulatory, copulation, and postcopulatory behavior and their duration were recorded for analysis. Moreover, the female’s body side approached by the male to move towards the posterior side of the female, as well as the side chosen by the male to turn 180° to allow end-to-end genital linkage and attempt the copula were recorded to understand the role of lateralized behaviors during *A. japonicus* mating. Unmated replicates were discarded before analysis.

### Mating frequency of female and male *A. japonicus*

A newly emerged virgin female (< 24 h old) was introduced to a Petri dish (diam. = 6 cm), and then a virgin male of same age was introduced to observe them continuously for 30 min. The mating times (i.e., frequencies) were recorded. If the male was still courting or copulating at the end of the observational period, observation will be continued until the male ceased courtship or a successful copulation was completed. After 30 min exposure, the female and male were separately isolated in a Petri dish (diam. = 6 cm). Female was provided with and without host eggs of *A. pernyi* for parasitization on daily basis to study multiple mating behavior of female and 20% honey solution as a food source to understand weather female re-mate at the state of diminished male sperm while the male was provided with only honey solution to carry out the experiment. Thereafter, the mated female was exposed to a newly emerged virgin male or mated male in a Petri dish at different time intervals (Table 3). The paired female and male were observed for another 30 min as described above. Male was removed from the Petri dish after 30 min observation. Mating times (i.e. frequencies) and durations were recorded for analysis. Further we also tested the multiple-mating behavior of mated male whether the male preferred to mate more than once or not (Table 4). Moreover, the starved (water fed) or honey-fed mated male was exposed to a new virgin female in a Petri-dish (diam. = 6 cm) at the different time intervals to observe the impact of diet on mating.

**Table 3:** Mating frequency treatment setup for *A. japonicus* females at different time intervals.

Mating status of female < 6 h*	Presence of host eggs (Yes or No)	Introduction of unexperienced virgin or mated male at different time intervals							
		6 h	12 h	24 h	48 h	5d	10d	20d	30d
Mated	Yes	Virgin	Virgin	Virgin	Virgin	Virgin	Virgin	Virgin	Virgin
Virgin	Yes	Virgin	Virgin	Virgin	Virgin	Virgin	Virgin	Virgin	Virgin
Mated	Yes	Mated	Mated	Mated	Mated	Mated	Mated	Mated	Mated
Mated	No	Virgin	Virgin	Virgin	Virgin	Virgin	Virgin	Virgin	Virgin
Virgin	No	Virgin	Virgin	Virgin	Virgin	Virgin	Virgin	Virgin	Virgin
Mated	No	Mated	Mated	Mated	Mated	Mated	Mated	Mated	Mated

*Females were divided in three categories based on the age; younger wasps (0 h–48 h old); mid age wasps (5–10 d); old wasps (11–30 d).

**Table 4:** Mating frequency treatment setup for *A. japonicus* males at different time intervals.

Mating status of male < 6 h*	Introduction of unexperienced virgin female at different time intervals				
	6 h	12 h	24 h	48 h	72 h
Virgin	Virgin	Virgin	Virgin	Virgin	Virgin
Honey fed mated	Virgin	Virgin	Virgin	Virgin	Virgin
Starved mated	Virgin	Virgin	Virgin	Virgin	Virgin

*Males were divided in three categories based on the age; younger wasps (0 h–12 h old); mid age wasps (48 h); old wasps (72 h).

### Effect of male body size on mating

Newly emerged males (< 24 h old) were selected randomly and kept in 1.5 ml vials separately to avoid fighting (self-observation). All male samples were put on ice in a container until they immobilized. Immediately after their immobilization, males were taken out from vials using a camel hair brush and their hind tibia lengths (H–L-T) were measured under stereomicroscope. Accordingly, sampled males were divided into two groups: large (H–L-T: no less than 0.063 mm), and small (H–L-T: no more than 0.044 mm). Here one large and small male alongside a newly emerged virgin female (< 24 h old) were introduced to a Petri dish (diam. = 6 cm). The selection of large or small male selected by female was observed through the naked eye until mating.

### Mating selection of virgin or mated male by female *A. japonicus*

Mating selection was observed via introducing virgin and mated male (< 24 h old) to a Petri dish (diam. = 6 cm). Fluorescent marking (FM) was done to differentiate among virgin and mated males and observed via ultraviolet (UV) light when needed. Then a newly emerged virgin female (< 24 h old) was introduced to the Petri dish. To check the impact of FM and UV the process was reversed on both virgin and mated males. Observation was made for 30 min to record successful rate of mating and percentage of virgin or mated males selected by female for mating.

### Statistical analysis

Whole data sets were checked for normality (Shaprio-Wilk) and homoscedasticity (Levene’s test) prior to analysis. Data having more than two groups and passed the test of normality was analyzed by one-way ANOVA (F), otherwise by Kruskal Wallis (H). Similarly, where two groups were compared and data met the normality assumption was analyzed by Independent sample T-test (*t*), otherwise by Mann Whitney (U) test. Laterality differences between the numbers of males approaching the left or right side of the female, as well as the number of males turning 180° to the left or to the right to attempt the copula during courtship interactions were analyzed using a Chi-square (χ^2^) test with Yates’ correction^64^. Proportion data was analyzed using generalized linear model Wald (χ^2^) considering binomial distribution (logit link function) and with least significant difference for pairwise comparison. All statistical analyses were performed using SPSS software (version 26) and figures were made in Origin Pro 2019.

## Data Availability

The data is available from corresponding author on reasonable request.
